# Using Economic Evaluation to Inform Responses to the Opioid Epidemic in the United States: Challenges and Suggestions for Future Research

**DOI:** 10.1080/10826084.2022.2026969

**Published:** 2022-02-14

**Authors:** Thomas Patton, Paul Revill, Mark Sculpher, Annick Borquez

**Affiliations:** a Division of Infectious Diseases and Global Public Health, University of California San Diego, California, USA;; b Centre for Health Economics, University of York, York, UK

**Keywords:** Economic evaluation, opioid use disorder

## Abstract

**Background:**

Several aspects of the opioid epidemic and of public health care organization in the United States (US) make the conduct of economic evaluation and the design of policies to respond to this crisis particularly challenging.

**Objectives:**

This commentary offers suggestions for how economic evaluation may address and overcome four key features of the opioid epidemic: 1) its magnitude and geographical distribution, 2) its intersection with multiple epidemics, 3) its rapidly changing dynamics, 4) its multi-sectoral causes and consequences.

**Results:**

We first offer pragmatic suggestions to address the difficulties in delivering a coordinated response given the fragmented nature of health care in the US. In view of the broad suite of responses required to address opioid use disorder and its associated comorbidities, we highlight the need for economic evaluations which consider interventions throughout the continuum of care (i.e. primary, secondary and tertiary levels of prevention). We examine how the use of predictive modelling alongside economic evaluation might be adopted to address the rapidly evolving situation affecting distinct populations and geographic areas and encourage investments in epidemic preparedness. Finally, we propose methods to capture the interdependence of various sectors of government affected by the opioid crisis in economic evaluations to ensure optimal levels of investment towards a comprehensive response.

**Conclusions:**

The opioid epidemic in the US represents an unprecedented public health challenge, but sound epidemiological modelling and economic analysis can help to guide use of limited resources committed to addressing it in ways that can have greatest impact in limiting its adverse consequences.

## Introduction

The shocking scale of the opioid epidemic in the United States (US) led it to being declared a national public health emergency in 2017 (Gostin et al., 2017). After falling for the first time in decades in 2018, drug overdose mortality started to rise in the months prior to the onset of the COVID-19 pandemic (Jalal & Burke, 2021) and has maintained its exponential increase since (Centers for Disease Control and Prevention, 2020). There were over 100,000 deaths in the 12 months up to May 2021, the highest number ever recorded over such period (Centers for Disease Control and Prevention, 2021, 2022). Establishing a timely and effective response is vital, but requires difficult choices to be made in the context of resource-constrained public health environments. Economic evaluation studies can elucidate this matter by weighing up the costs and benefits of alternative policy responses in order to guide the allocation of resources toward policies that are expected to deliver maximum improvements in population health. However, particular features of the opioid epidemic make the conduct of economic evaluation and the design of policies particularly challenging, especially given the organization of health care in the US. In this paper, we examine four features of the opioid epidemic which pose challenges to economic evaluation and to the design of effective policies. We offer suggestions to guide future research efforts supporting this endeavor.

### Challenge 1: The magnitude and geographical distribution of the opioid epidemic across fragmented health care systems

The opioid epidemic has claimed over 840,000 drug overdose deaths between 1999 and 2020 (Centers for Disease Control and Prevention, 2020). When confronted with a crisis of this magnitude, the government would typically be the natural leader for coordinating a response of investments distributed across the territory according to epidemic burden. However, the formulation of a national coordinated response informed by economic evaluations would be challenging, not least because there is a history of political resistance to the mandated use of cost-effectiveness criteria from the federal government (Neumann, 2004; Chambers et al., 2015). This is perhaps most notably exemplified by the so-called “non-interference clause” which banned price negotiations between Medicare and pharmaceutical companies (Lee et al., 2016). Second, the fragmented provision of health care between multiple public (Medicare, Medicaid) and private systems in the US creates logistical problems in conducting economic evaluations and in implementing public health programs accordingly. To be reliably informative for policy responses, economic evaluations should incorporate all relevant evidence to inform estimates of parameters relating to clinical effects, health-related quality of life and unit costs (Sculpher & Drummond, 2006). Given that these parameters are likely to vary across payers and geographic regions, this raises the question as to whether separate economic evaluations need to be developed for all the respective decision makers and geographical levels or if analyses can simultaneously inform decisions across the whole country (Ederhof, 2021). These issues would arguably be best managed by a central body responsible to oversee policy making research efforts, such as the identification and synthesis of evidence, and to deliver region- and payer-specific evaluations. Many countries have organizations responsible for the conduct of health technology assessments, which include economic evaluations, to guide the allocation of resources in their health care systems (Drummond, 2013).

The use of economic evaluation to inform resource allocation decisions in the US is highly fragmented, especially when compared to countries such as the UK, France or Germany. For instance, economic evaluations are used by the National Institute for Health and Care Excellence to inform the resource allocation decisions in the single-payer healthcare systems in England and Wales (Thokala et al., 2020). One agency that has received attention for producing comprehensive health technology assessments (including economic evaluations) in the US is the Institute for Clinical and Economic Review (ICER) (Pizzi, 2016). ICER is an independent, nonprofit organization that aims to support health payers in the US in making decisions about pricing and access of health interventions through its assessments. In the context of OUD, ICER has reviewed a range of medications, including reformulated prescription opioids (to prevent use other than as prescribed) and medications for OUD (MOUD), as well as recent assessments of supervised consumption rooms and digital apps to assist outpatient treatment of OUD (Institute for Clinical and Economic Review (ICER), 2020). Although ICER may be highly influential in terms of its evidence-based recommendations, it has no formal affiliation with any of the Government-funded health care programs and therefore does not directly inform the use of specific interventions by these programs. Importantly, creating formal collaborations between ICER, research institutions and Government-funded health care programs would encourage greater coordination and data sharing in the synthesis of evidence and development of models for the evaluation of alternative policy responses. Given the reluctance of the federal government to endorse the use of cost-effectiveness research for policy-making, this would require an explicit commitment from the government to both recognize and allocate resources toward the need for cost-effectiveness research. This would also require the removal of obstacles such as the “non-interference clause” in the case of Medicare (Lee et al., 2016). This type of deliberative process would, in turn, facilitate more robust decision making and intervention implementation across the country.

While the case for ICER having a stronger mandate in health care decision-making is not unique to OUD, it is particularly salient due to the high proportion of care for OUD that is provided by Government-funded programs and the large number of interventions requiring evaluation. Another strategy to achieve an evidence-based and large-scale response would be through focusing on key players in the response to the opioid epidemic. Medicaid government assistance programs that fund health care coverage for eligible low-income individuals and vary from state to state, could benefit substantially from economic evaluations given the considerable population health and financial burden that the treatment of OUD and co-occurring mental health disorders places upon them (Leslie et al., 2019). However, to date, only a handful of studies have explicitly sought to investigate the cost-effectiveness of OUD interventions from the perspective of a state Medicaid program (Beaulieu et al., 2021; Onuoha et al., 2021). In addition to state Medicaid programs, local health departments (LHDs), which are responsible for surveillance and prevention activities, play a crucial role in responding to opioid use and overdose within their own communities (often at county-level) (Evans et al., 2019). They can act at many levels, including through information dissemination campaigns (to prevent the non-prescribed use of opioid pain relievers, to increase overdose risk awareness, to reduce stigma against OUD), through the implementation of drug take back and naloxone distribution programs, and through the coordination of multiple governmental and non-governmental organizations to improve linkage to appropriate health and social services in the community (Raja et al., 2019). However, their resources are tightly limited and guidance on how to effectively allocate them toward specific interventions and population groups is much needed. Developing generic economic models that can be applied at the LHD level across states, fully incorporating local epidemiological data (Raja et al., 2019) would provide a rigorous framework to inform decision-making. Capacity constraints mean that most LHDs are in no position to accomplish this task independently (Teutsch & Fielding, 2016) and these initiatives require support to be coordinated.

### Challenge 2: The intersection of multiple epidemics

The progressive and relapsing nature of OUD, and the multiple health consequences associated with it, implies that interventions are required at multiple levels, from prevention to harm reduction and from mental health treatment to wound care. Typically, interventions for OUD are classified into three levels (Kolodny et al., 2015): primary-level interventions are intended to prevent new cases of OUD from occurring, e.g. drug use education campaigns, mental health care (Compton et al., 2019; Koh, 2017); secondary-level interventions are concerned with screening for OUD after its onset but before causing serious complications, e.g. prescription drug monitoring programs (Thomas et al., 2014); and tertiary-level interventions for individuals whose OUD is firmly established, such as MOUD (e.g. methadone or buprenorphine) (Larney & Hall, 2019) and harm reduction approaches such as drug testing services, syringe services programs and naloxone distribution (Rouhani et al., 2019). Using models that capture the knock-on and long-term impacts of upstream and downstream interventions provides a more accurate picture of the economic value of interventions requiring large scale and long-term investments (Tappenden et al., 2012). In parallel, multiple co-morbidities, including infectious diseases such as HIV, hepatitis C (HCV), and tuberculosis, as well as chronic diseases such as congestive heart failure, are more prevalent among people with OUD (Bahorik et al., 2017). Covid-19 has also been viewed as an intersecting epidemic due to increased severity among people with OUD and to multiple pandemic stressors being conducive to drug use (Khatri & Perrone, 2020; Schimmel & Manini, 2020). Multiple studies have examined the spread of infectious diseases in populations affected by OUD, particularly the comparison of different combination of interventions to reduce the incidence of specific infections such as HIV or HCV (Bernard et al., 2017; Cousien et al., 2015; Fu et al., 2018). While these studies have traditionally focused on individual diseases, recent initiatives have aimed to consider intersecting epidemics (Bórquez et al., 2020; Cepeda et al., 2020), which could be key to informing integrated healthcare for OUD and other substance use disorders. These developments are particularly important in view of recent claims that a failure to account for comorbidities in a model may threaten the validity of its outputs (Guthrie et al., 2017). This implies a need to move away from models that evaluate interventions in a piecewise approach (e.g. interventions for specific health harms such as overdose, HCV or HIV alone) to those capable of evaluating multiple outcomes.

Model-based economic evaluations concerned with multiple comorbidities are inherently more complex than those for single health conditions (Guthrie et al., 2017). Not only do these models need to account for the natural histories of separate conditions but they must also contend with any interactions between them, as well as between their treatments. For example, Cepeda and colleagues developed a dynamic model of HIV transmission and overdose among people who inject drugs (PWID) in Tijuana to assess the impact and synergies of scaling-up integrated antiretroviral therapy (ART) and MOUD (Cepeda et al., 2020). Another study by Birger and colleagues showed that scaling up HCV treatment alongside existing ART and MOUD program efforts among PWID in Vietnam would achieve substantial reductions in mortality on account of the decreased incidence of cirrhosis and liver cancer among those on ART (Birger et al., 2017). Accounting for these synergies has implications in economic evaluations as MOUD is shown to be more cost-effective if we incorporate its effects on multiple diseases and treatment engagement and, similarly, HCV treatment is shown to be more cost-effective in the context of ART scale up. Important lessons on this matter can also be drawn from work by Guthrie and colleagues (2017), who investigated the cost-effectiveness of pharmacological interventions in patients with depression and at-risk of coronary heart disease. This work illustrated the important role that expert elicitation exercises can play in formulating model assumptions where evidence on interactions between conditions is lacking.

### Challenge 3: The rapidly changing dynamics of the opioid epidemic

The economic evaluation of interventions to address OUD and co-morbidities is further complicated by the rapidly evolving nature of the opioid epidemic, which has been described as a four-wave epidemic (Ciccarone, 2019; 2021). The first wave started with the prescription opioid epidemic in the early 2000s, reaching previously unaffected rural and suburban areas. This was followed by a heroin epidemic in 2010, associated with higher risk of infectious disease transmission through injecting drug use. The third wave began in 2013 through the illegal supply of fentanyl and other synthetic opioids, leading to extremely high increases in overdose deaths due to its high potency. More recently, a fourth wave of stimulant-involved overdose deaths has been documented resulting from polydrug use (use of stimulants, such as methamphetamine or cocaine, combined with opioids). These epidemics and associated health harms are modulated by variations in local drug markets and by different patterns of polydrug use, affecting different demographic groups in distinct geographical areas, leading to a constant evolution.

Economic evaluations of public health strategies to address chronic diseases often use static models based on a known disease prevalence and a constant exposure to risk factors. In some cases, these models might be ill-suited to inform the response to the opioid epidemic (or more exactly, to its sub-epidemics) as they might under-estimate the cost effectiveness of implementing specific prevention interventions by not accounting for the risk of outbreaks occurring. Sudden increases in the incidence of OUD, polydrug use or in the geographic scope of specific drugs (i.e. changes in the exposure to the risk factor) would be better addressed using predictive statistical or dynamic models that incorporate cost-effectiveness analyses (Marks et al., 2021a). Efforts in this space are emerging and statistical models have been developed to predict the incidence of OUD and the associated drug use harms in the near future (i.e. next month or year) to identify key counties in need of prevention interventions (Campo et al., 2020; Marks et al., 2021b; Sumetsky et al., 2021; Van Handel et al., 2016).

Alongside these predictive models, economic evaluations to inform investments to mitigate drug use epidemics before they occur, akin to those implemented in the context of natural disaster risk reduction (Idris, 2018; Vorhies, 2012), would further guide and strengthen epidemic preparedness. A more fundamental challenge in these predictive endeavors, which also directly affects economic evaluations, lies in the lack of reliable data on the true prevalence and incidence of OUD. Studies have shown that opioid use and OUD prevalence estimates obtained through the national drug use and health survey (NSDUH) are likely greatly underestimating true prevalence (Barocas et al., 2018) and therefore predictive models often rely on proxy measures of prevalence (e.g. overdose mortality rates, opioid-related hospitalizations). Robust estimates of the size of the population with OUD in a given jurisdiction are necessary for estimating the expected budget impact of introducing an intervention. A promising method to indirectly estimate OUD prevalence, that has yet to be utilized in the US context, involves the use of individual-patient overdose mortality data in combination with aggregated population overdose mortality data (Jones et al., 2020).

In addition to better opioid use and OUD prevalence data, preparedness in the context of large inflows of synthetic opioids or other drug market changes entails the development of stronger surveillance systems monitoring their related health harms. Coroner death scene investigation systems are an especially important tool for the early detection and prediction of drug overdose outbreaks (Boslett et al., 2020; Williams et al., 2017). Sadly, there have been reports that medical examiner and coroner offices are “facing overwhelming caseloads that require more complex and expensive investigations” (Ropero-Miller et al., 2020). This has led to calls for greater investment in infrastructure to achieve timely data reporting (Fliss et al., 2021). Similarly, enhanced infectious disease surveillance to enable the early identification of substance use related HIV, HCV, tuberculosis, or other infectious disease outbreaks is warranted. Ascertaining the economic value of strengthening surveillance systems in the context of substance use could be key to advocating for these investments among policy makers.

In addition to improving surveillance, investing in the drug use treatment and harm reduction infrastructure to ensure high coverage of MOUD, syringe exchange, and naloxone distribution programs is key to outbreak prevention. The constant evolution of the epidemic also means that novel intervention approaches might be needed to respond to the specific public health challenges. For example, drug testing strips and low threshold supervised consumption services (in tents or other temporary spaces) appeared as emergency harm reduction tools with the emergence of fentanyl. Given that evidence may be sparse for resource allocation decisions in the context of emerging substance use outbreaks, an iterative approach may be warranted with early health economic modeling techniques used in the initial stages to understand the key drivers of cost-effectiveness and to inform future research needs (Drummond, 2020).

Evaluations to estimate the economic costs of substance use related outbreaks can reveal the gamble made by specific counties or states when foregoing potentially critical investments in both surveillance and prevention. As such, this type of evaluation could provide timely guidance on both epidemic preparedness investments and the scale up of (potentially novel) emergency interventions (Rosenblum et al., 2020).

### Challenge 4: The multi-sectoral causes and consequences of opioid use disorder

Beyond OUD being a complex condition necessitating a myriad of healthcare interventions, a response above and beyond their scale up is required to address the structural drivers and consequences of the crisis (Parker et al., 2019; Wakeman et al., 2014). There is growing recognition that the root causes of OUD are a combination of economic and social factors, i.e. wage stagnation, unemployment, a lack of affordable housing, hopelessness and despair (Dasgupta et al., 2018). While there have been calls to address these driving factors through investments to promote access to education, employment, welfare and housing and to reduce drug use criminalization and stigma and foster community cohesion, the evidence base supporting the cost-effectiveness of for these types of interventions is limited (Park et al., 2020). Economic evaluations of these broader types of social programs could prove vital in establishing the political buy-in required for their implementation.

As previously mentioned, the opioid crisis has also inflicted its own socio-economic harms. Between 2000 and 2016, opioid-related reductions in labor market participation were estimated to cost state and federal governments $36.1 billion in lost income tax revenue (Segel et al., 2019a). Labor market exits also lead to greater demands being placed on means-tested social programs such as cash assistance schemes and unemployment benefits (Segel et al., 2019b). The costs to the criminal justice system associated with OUD in 2017 were estimated to be $14.8 billion (Florence et al., 2021). Between 2011 and 2016, caseloads involving the children and adolescents whose parents were affected by OUD were estimated to cost the US Child Welfare $2.8 billion (Crowley et al., 2019). Existing studies in OUD illustrate that non-health costs can be incorporated in economic evaluations (Beaulieu et al., 2021; Onuoha et al., 2021). However, these studies have tended to aggregate health and non-health costs which can be misleading for decision makers aiming to make optimal use of health care budgets if there is no scope for compensation from other sectors benefiting from a given reimbursement decision. Ideally, resource allocation decisions would involve decision makers acting on behalf of the different sectors to achieve mutually beneficial investments, where investments are commensurate to the benefits accruing from the decision in each sector (Walker et al., 2019).

A recent paper illustrates how this type of exercise might be conducted and presents a case study of a cost-benefit analysis (CBA) of an OAT program in Armenia (Stuart & Wilson, 2020). This study estimated the proportion of benefits realized from reductions in HIV infections relative to the total benefits of the program (i.e. reduced risks of HCV infection, opioid overdose, financial stress, and crime) and showed that this implied 48% of the program costs, at most, should come from budgets earmarked for HIV interventions. CBA attempts to monetize both costs and benefits of policy alternatives and so appears to be straightforward from an aggregation standpoint. Stuart and Wilson (2020) estimated the total monetary benefits of OAT based on an assumed benefit-cost ratio (based on estimates from previous studies) combined with an estimate of the cost of implementing the program. Despite its intuitive appeal, there have been serious questions posed as to whether the techniques used to measure benefits in monetary terms can provide an “acceptable representation of public interest” (Culyer & Chalkidou, 2019). In the absence of a validated framework for comparing valuations of outputs of health care interventions and those in other sectors, a solution recommended by Walker and colleagues, is to disaggregate costs and consequences by sector in economic evaluations (Walker et al., 2019). The disaggregated method ensures that the broader costs and consequences are reported, which may then permit negotiations amongst decision makers for various sectors.

Another recent study demonstrates the implementation of this approach in an economic evaluation of interventions for alcohol use disorder in the UK (Ramponi et al., 2021). In addition to presenting health gains in terms of QALYs, this study estimated the benefits of reductions in crime both in terms of reduced recidivism rates and reduced QALY losses for the victims of those crimes. Critically, the results showed that the recommended intervention differed depending on the perspective adopted (i.e. health, criminal justice, or both) and the normative value judgements used to inform decisions. Future research is needed to reproduce this approach in economic evaluations for OUD to ensure the appropriate allocation of resources toward OUD interventions. Of course, there may be more fundamental policy evaluations to consider in the allocation of resources to criminal justice system given the long-standing criticisms of mass incarceration and drug criminalization policies (Travis et al., 2014).

## Conclusion

This commentary describes main challenges and offers suggestions for future research to guide responses to the opioid epidemic in the US, as summarized in Table 1. First, we look at the magnitude of the opioid epidemic and how the fragmented nature of health care in the US makes a coordinated response challenging and recommend the creation of formal collaborations between ICER and government funded health programs (i.e. Medicaid) to implement economic evaluations of health technologies addressing OUD. We also recommend a pragmatic approach from researchers to address the heterogeneity in epidemic patterns and responses across the country, namely the development of generic economic models and their application to inform resource allocation by State Medicaid organizations and local health departments. Second, we describe the multiple comorbidities associated with OUD and the broad suite of responses needed. We recommend implementing an integrated health care perspective, which considers the multiple health outcomes of OUD and appropriate interventions at primary, secondary and tertiary levels to identify optimal intervention packages. Next, we consider the rapidly evolving situation of drug use epidemics, affecting distinct populations and geographical areas over time, and highlight the need for establishing real time surveillance of drug use patterns and developing predictive dynamic models and risk assessment economic models which allow the economic evaluation of investing in a preemptive response. Finally, the multi-sectoral causes and consequences of the opioid crisis call for government agencies operating in different sectors to coordinate the allocation of resources. We therefore call for a disaggregated presentation of costs and consequences by sector to facilitate negotiations amongst decision makers for various sectors. The opioid epidemic in the US represents an unprecedented public health challenge, but sound epidemiological modeling and economic analysis can help to guide the use of limited resources committed to addressing it in ways that can have greatest impact in limiting its adverse consequences.

## Figures and Tables

**Table 1.** Recommendations for future research and policy involving economic evaluations to guide responses to the opioid epidemic in the US.

Challenge 1	The magnitude and geographical distribution of the opioid epidemic across fragmented health care systems
Recommendation 1	Need for a central body with a formal mandate to coordinate research efforts in the conduct of economic evaluations of health technologies addressing OUD (e.g. medications), to inform decisions among government funded programs across the whole country.
Recommendation 2	Pragmatic approach from researchers considering critical areas, namely the development of generic models and their application to inform resource allocation by state Medicaid organizations and local health departments.
Challenge 2	The intersection of multiple epidemics
Recommendation 1	Implement an integrated health care perspective with “whole disease” models of OUD, which consider disease manifestations at different stages of progression and interventions at primary, secondary and tertiary levels to identify optimal combination.
Recommendation 2	Build on methodological research to develop models concerned with the multiple comorbidities (i.e. OUD, mental health disorders, HIV, and HCV).
Challenge 3	The rapidly changing dynamics of the opioid epidemic
Recommendation 1	Further research and implementation of new methods for indirectly estimating OUD prevalence (e.g. capture-recapture and methods which combine data from multiple sources).
Recommendation 2	Integrate economic evaluation with predictive modeling approaches from epidemiological research to evaluate surveillance systems, epidemic preparedness investments, and the scale up of emergency interventions.
Challenge 4	The multi-sectoral causes and consequences of opioid use disorder
Recommendation 1	Economic evaluations of upstream/structural programs/interventions to address economic and social determinants as the root cause of OUD are needed.
Recommendation 2	Disaggregated presentation of upstream program costs and consequences by sector to facilitate negotiations amongst decision makers for various sectors.
